# Shared decision-making in end-of-life care for end-stage renal disease patients: nephrologists’ views and attitudes

**DOI:** 10.1186/s13584-024-00632-w

**Published:** 2024-09-10

**Authors:** Wassiem Bassam Abu Hatoum, Daniel Sperling

**Affiliations:** 1 Faculty of Social Welfare and Health Sciences, Department of Nursing, University of Haifa, 199 Aba Khoushy Ave. Mount Carmel, Haifa, Israel; 2 Department of Nephrology and hypertension, Haemek Medical Center, Afula, Israel Yitshak Rabin Boulevard , 1834111

**Keywords:** End-of-life, Shared decision-making, End-stage renal disease, Nephrologists, Ethics

## Abstract

**Background:**

The term *end-stage renal disease* (ESRD) refers to the final stage of chronic kidney disease. Not all ESRD patients are suitable for dialysis treatment, which despite its advantages, is not without risks. Shared nephrologist-patient decision-making could be beneficial at this stage, yet little is known about such practices in Israel. This study aimed at examining the practice of shared decision-making (SDM) between nephrologists and ESRD patients in Israel, while exploring related conflicts, ethical dilemmas, and considerations.

**Methods:**

The descriptive-quantitative approach applied in this study included a validated questionnaire for nephrologists, based on Emanual and Emanual (1992). The survey, which was distributed via social-media platforms and snowball sampling, was completed by 169 nephrologists. Data analysis included t-tests for independent samples, f-tests for analysis of variance, and t-tests and f-tests for independence. Descriptive analysis examined attitudes towards SDM in end-of-life care for ESRD patients.

**Results:**

The findings show that the research sample did not include nephrologists who typically act according to the *paternalistic* decision-making style. Rather, 53% of the respondents were found to act in line with the *informative* decision-making style, while 47% act according to the *interpretive* decision-making style. Almost 70% of all respondents reported their discussing quality-of-life with patients; 63.4% provide prognostic assessments; 61.5% inquire about the patient’s desired place of death; 58.6% ask about advance directives or power-of-attorney; and 57.4% inquire about cultural and religious beliefs in end-of-life treatment. Additionally, informative nephrologists tend to promote the patients’ autonomy over their health (*P* < 0.001); they are also in favor of conservative treatment, compared to paternalistic and interpretive nephrologists, and use less invasive methods than other nephrologists (*P* = 0.02).

**Conclusions:**

Nephrologists in Israel only partially pursue an SDM model, which has the potential to improve quality-of-care for ESRD patients and their families. SDM programs should be developed and implemented for increasing such practices among nephrologists, thereby expanding the possibilities for providing conservative care at end-of-life.

## Introduction

The term *end-stage renal disease* (ESRD) refers to the final stage of chronic kidney disease [1]– including transplantation, hemodialysis, and peritoneal dialysis [2]. Yet dialysis is not simple to endure, especially as most patients are elderly and have complex medical backgrounds [3]. Dialysis may also have undesirable psychosocial outcomes, and its aims for a specific patient may not always be clear [4]. As such, not all patients are suitable for dialysis, and those who do embark on this treatment may become too frail to continue [5]. In such cases, more conservative treatment may be considered a better alternative, such as pain control, psychological and emotional therapy [6], and even palliative care (PC) – especially when the outcomes of prolonged survival do not seem beneficial [4, 7]. In light of this complexity, guidelines have been issued in the USA for treating ESRD patients, including the addressing of issues such as futile dialysis, withdrawing dialysis, and PC [8–10].

These guidelines also highlight the importance of shared nephrologist-patient decision-making – a process that would help nephrologists deal with related dilemmas. In *shared decision-making* (SDM), both the healthcare professional and the patient play a role and make decisions regarding the course of treatment that will be pursued [11]. Moreover, the approach of *inter-professional SDM* entails collaborations between a number of healthcare professionals and the patients themselves, with the aim of making optimal decisions that also consider the patient’s personal preferences [12]. Encouraging such collaborations between multi-disciplinary teams, while allocating team tasks, could play a pivotal role in integrating and maintaining SDM as a routine medical practice [13].

According to the American Renal Physicians Association, SDM is especially desirable when caring for ESRD patients [14]. Yet little is known about SDM and end-of-life (EOL) care among nephrologists in Israel. Since related guidelines in Israel do not exist, this study is of great importance – especially as physicians have been found to make treatment-related decisions based solely on the patient’s age and comorbidity [15]. The aim of this study, therefore, was to examine the degree to which SDM is applied when treating ESRD patients in Israel, while exploring ethical and other dilemmas that nephrologists face when providing such patients with EOL care.

## Literature review

### Shared decision-making (SDM)

SDM enables clinicians and patients to jointly choose the treatment path, after assessing the options and considering the patient’s preferences [16, 17]. SDM strives to prioritize the patient [12], by addressing their preferences, improving their knowledge, and enhancing clinician-patient communications [17]. Yet in Israel, nephrologists lack practical, research-based guidelines for conducting SDM – in EOL care in general, and in ESRD patients in particular. Moreover, these physicians do not undergo adequate SDM training, nor do they have sufficient access to the relevant research literature [17]. Such guidelines are especially important as nephrologists may suffer emotional burden following their decision-making, for example when a patient does not fare well with dialysis, yet the alternative is imminent death [18, 19].

While SDM has become a common clinical practice in nephrology care in the USA [20, 21], and a health-policy priority in Europe [16], it has not been broadly or officially introduced into nephrology units in Israel. More generally, SDM is not frequently reported in Israel. First, patients tend to rely on their physicians to make the right decisions for them; alternatively, physicians may implement persuasion tactics, to encourage the patient to agree with the course of action that they have suggested. Moreover, physicians lack training in SDM, especially during their medical preparation. As a result, they may not adequately understand or interpret the risks and benefits that are characteristic of SDM [22].

### Barriers in caring for ESRD patients

In the USA, variations can be seen in decision-making practices among nephrologists when providing EOL care [7, 8, 23]. Medical directors report that they respect the requests of their competent patients to withhold or withdraw from dialysis; 17% agree that they would start or continue dialysis in permanently-unconscious patients; and 32% would do so for patients with advanced dementia or without advance medical directives [24]. Nephrologists also claim that their dialysis-related decisions are most influenced by their patients’ preferences and by the clinical urgency, followed by input from family members [19, 25, 26]. Finally, older nephrologists (≥ 65 years) are more likely to recommend dialysis rather than conservative care (CC) compared to their younger colleagues [27].

Studies conducted in the USA found that most dialysis professionals conduct EOL discussions with patients and feel well prepared for making related decisions; they also prefer to follow a decision-making model, and tend to provide recommendations after presenting the patient with various treatment options [15]. Most nephrologists report that they feel comfortable with providing EOL care for patients who have advanced chronic kidney disease [19, 26, 28, 29]; yet they do face a range of challenges when discussing EOL with their patients, such as the families’ lack of cooperation and their own fear of eliminating hope. Additional barriers stem from inadequate palliative or hospice care options, and from the family’s reluctance to discuss EOL care [19, 28]. Other reasons may include the patient’s refusal to participate in the decision-making process, difficulty understanding the treatment options, and lack of patient motivation. Decisions may be made quickly, without weighing all of the options that are available to the patient. In some cases, the clinician’s decision-making style inhibits SDM – especially when lacking SDM-specific training or supporting services [30].

SDM may not always be applied in cases where the patients explicitly state their desire not to pursue dialysis, yet the physicians disagree with their choice – especially if they are not certain of their patients’ competency to make such decisions. From the patients’ point-of-view, feelings of frustration or hostility may arise if the physician repeatedly questions the patients’ preferences. Patients may even could comply or tolerate dialysis, despite their preference to withhold such treatment, when physicians convince them that this is a temporary procedure [31]. Finally, seriously-ill patients may struggle with their need to participate in emotionally complex discussions regarding their prognosis and treatment options, rendering them unable or unwilling to make decisions [32].

To the best of our knowledge, EOL care in ESRD patients has not been investigated in Israel from the nephrologists’ perspective. A recent study examined the nephrology nurses’ views of SDM practices among the nephrologists with whom they work [33]. In another study, family physicians perceived AD as a complex issue and lacked knowledge on PC [34, 35]. Physicians have stated that they believe their EOL patients have the right to make decisions regarding life-prolonging treatment, yet some also claimed that such patients often receive unnecessary treatment [34, 36]. In one study, about half the physicians deemed expensive treatments in EOL care unnecessary [36]. Some reported that they refrain from talking to their patients about their prognosis, or about the option of withdrawing treatment, simply focusing on providing PC instead. On the other hand, some physicians may succumb to the family’s demands to provide life-saving (and possibly futile) treatment [34, 35, 37, 38].

### Nephrologists and SDM

When conducting informed SDM regarding renal-replacement therapy [39], the nephrologist’ role is pivotal. The literature defines four different approaches to such decision-making processes: [1] *Paternalists*, who prioritize their patients’ health over their patients’ decision-making autonomy; [2] *Institutionalists*, who treat their patients according to the institution’s norms and culture; [3] *Informativists*, who prioritize their patients’ decision-making autonomy; and [4] *Interpretivists*, who develop strong ties with their patients, as a means for facilitating guided decision-making [40]. In general, the first two types of nephrologists (paternalists and institutionalists) would favor dialysis in most cases, while the latter two (informativists and interpretivists) focus on the patients’ engagement and their quality-of-life.

In the USA, nephrology medical directors encourage their teams to apply SDM for initiating or withdrawing dialysis, in line with the guidelines issued by the American Renal Physicians Association [19]. Yet when treating ESRD patients, nephrologists must overcome SDM-related concerns, in order to adequately convey the various options to their patients. Nephrologists with positive attitudes towards dialysis tend to report fewer barriers [41]. Although SDM has not been investigated in the field of nephrology in Israel, it has been researched in other medical fields [42–45]. Studies show that older, more experienced physicians usually address caregivers rather than the patients themselves; primary-care physicians also tend to make decisions together with the patient’s family [46]. Overall, SDM in Israel is perceived as feasible, and could lead to increased engagement and knowledge, improved medical outcomes, and better correspondence with the patients’ preferences [47].

## Methods

### Conceptual framework

**Fig. 1:**
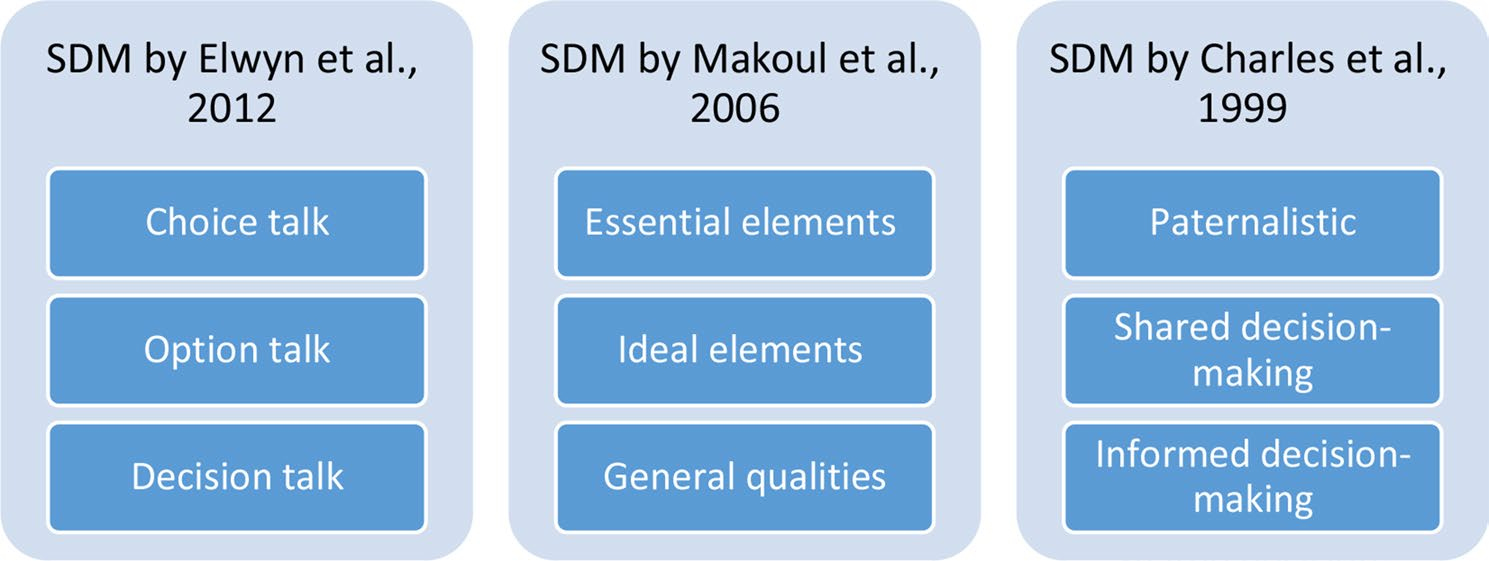
Conceptual Research Framework

The conceptual framework applied in this research study combines the following three theoretical SDM models: [1] practical SDM steps, including talking about choice, options, and decisions [48]; [2] SDM categories, including essential, ideal, and general SDM qualities [49]; and [3] three types of decision-making, including paternalistic, SDM, and informed decision-making [50] (see Annex 2 and Fig. 1). The research approach applied in this study included questionnaires for nephrologists, based on the theory developed by Emanual and Emanual (1992) and on the four models of physician-patient relationships (paternalistic, interpretive, informative, and deliberative) [51], as discussed above.

### Research hypotheses

Based on the literature review and the conceptual framework presented above, we propose the following two hypotheses:

H1: Nephrologists who act according to the interpretive and informative decision-making styles, and nephrologists who work in public or government dialysis clinics, will tend to support conservative treatment and focus on patient engagement, values, autonomy, and quality-of-life – compared to other respondents.

H2: Nephrologist who act according to the paternalistic decision-making style, and older nephrologists, will tend to prioritize patient health over autonomy, and be in favor of initiating dialysis as a measure of success – compared to other respondents.

### Research design

This study applied a descriptive-quantitative approach for examining the perceptions of nephrologists in Israel in relation to SDM in ESRD patients. To do so, a cross-sectional descriptive questionnaire was compiled, based on the literature and on the theoretical models presented above (Annex 1).

### Research population, sample, and sampling

Snowball and purposive sampling methods were employed to recruit respondents. The questionnaires were completed by 169 nephrology physicians. (According to official data from the Israeli Society of Nephrology and Hypertension (ISNH), this number constitutes about 60% of the nephrologist population in Israel.) Forty respondents completed the questionnaire in writing, while 129 completed it online. The respondents included nephrology physicians from hospitals and from community-based dialysis clinics throughout the country. Table 1 describes the research sample.

**Table 1:** Respondents’ characteristics

Variable	Findings
Gender	Male = 53.5%; Female = 46.5%
Age	26–75 years (M = 50, SD = 11)
Country of birth	Israel = 70.6% Russia = 14.1% Argentina = 3.7% Other countries = 11.6%
Marital Status	Married = 78.7% Single = 10.3% Divorced = 6.5% In a relationship = 2.6% Widowed = 1.9%
Nationality	Jewish = 71.7% Arab = 28.3%
Religion	Jewish = 71.7% Christian = 10.1% Muslim = 18.2%
Level of Religiosity	Ultra-Orthodox = 7.9% Religious = 32.2% Traditional = 24.4% Secular = 35.5%
Medical Specialization*	Nephrologists = 81.7% Nephrologists and Palliative Care = 18.3%
Work Experience	16 (average); 0–40 (range); 10 (SD).
Workplace	Public Hospital 78.7%; Governmental Hospital 23.7%; Private Hospital 8.9%, Community Private Dialysis Clinics 16.6%.
Frequency of exposure to fragile patients	> 30% meet ESRD patients aged 75 and older about 10–20 times a month; 19% encounter patients with sepsis; 14% care for patients with advanced stages of dementia; 10–12% care for patients with other co-morbidities.
Area of workplace	South 13.6%; Center 44.4%; Jerusalem and surrounding area 18.9%; North 18.3%; HaSharon 1.8%.

* In Israel, prior to specializing in nephrology, physicians must be certified General Physicians

### Data collection

First, a pilot study of the comprised questionnaire was conducted, delivered by post and by email to five physicians from nephrology units in Israel. Following their feedback and insights, the questionnaire was revised as needed. Using the QualtricsXM software, the final questionnaire was then posted on a range of online platforms, including eight professional Facebook groups and three WhatsApp groups. The questionnaire was also sent via Listserv to members of the ISNH, through snowball sampling and direct requests to participate. Printed copies of the questionnaire were also handed out at staff meetings and at two national nephrology conferences.

### Data analysis

The data obtained from the questionnaires were analyzed using descriptive and inferential statistics. A range of associations and relationships between variables were examined in line with the conceptual background of this study, such as the respondents’ tendency to choose their patients’ health over their autonomy (or vice versa) and their age or years of experience in the field.

To test the research hypotheses, the following three primary variables were calculated: [1] Decision-making style. This variable was based on 27 items from the questionnaire, coded on a 1–5 scale (Cronbach’s α = 0.96). An average of all items was calculated and divided into three types: paternalistic (M ≤ 2), interpretive [2–4], or informative (M ≥ 4); [2] Health vs. autonomy. This variable was based on three items from the questionnaire, coded on a 1–5 scale (Cronbach’s α = 0.91). An average of all items was calculated. This variable was used as a continuous scale, whereby lower scores represented *health*, while higher scores represented *autonomy*; and [3] Conservative vs. invasive treatment. This variable was based on 16 items from the questionnaire, coded on a 1–5 scale (Cronbach’s α = 0.84). An average of all items was calculated and divided into two types: invasive (M ≤ 3) and conservative (M ≥ 3). We used five different statistical tests for data analyses: Pearson correlations; Fisher’s exact test; t-tests for independent samples; Chi-square test for independence; and t-tests for independent samples.

### Ethical considerations and approvals

This research study was approved by the Research Ethics Committee at the Faculty of Social Welfare and Health Sciences, University of Haifa (Approval #411/21, dated 13 July 2021). The respondents were informed that their participation was voluntary and that they could cease participation at any time whatsoever. A short description of the research aims, expected advantages, risks, confidentiality, expected duration for completing the questionnaire, and research funding were all presented on the first page of the questionnaire – after which the participants were asked to provide their informed written consent. Participation in the study was anonymous and complete confidentiality was maintained throughout the study. Additionally, all research tools and methods were applied in accordance with strict ethical standards, as published in the Declaration of Helsinki and its later amendments (or comparable ethical standards).

## Results

The findings of this study regarding SDM between nephrologists and ESRD patients in Israel are presented through descriptive and inferential statistics.

### Respondents’ characteristics

Out of the 169 participants, 40 respondents completed a printed version of the questionnaire and 129 respondents completed an online version via a link that was posted on professional Facebook and WhatsApp groups. When examining the respondents’ personal characteristics, the research sample included 53.5% males and 46.5% females, and the respondents’ mean age was 50 years (SD ± 11). Most respondents were born in Israel (70.6%) and were married (78.7%). When asked about their nationality, 71.7% defined themselves as Jewish and 28.3% as Arabs, including 18.2% Muslim-Arabs and 10.1% Christian-Arabs. When asked about their religiosity, about 35.5% of the respondents defined themselves as secular, 24.4% as traditional, 32.2% as religious, and 7.9% as orthodox.

From a professional perspective, all respondents were general physicians; 81.7% were nephrologists and 18.3% also specialized in PC. Some respondents had more than one place of occupation: 78.7% worked in public hospitals (including 23.7% in government hospitals) and 8.9% in private hospitals. Additionally, 16.6% worked in private community dialysis clinics, either part-time or full-time. Finally, 74.3% reported working in central Israel, 47.8% in the Jerusalem region, 57.4% in the north of Israel, 37.7% in the south, and 7.3% in the Sharon region (Table 1).

### SDM practices and perceptions

**Fig. 2:**
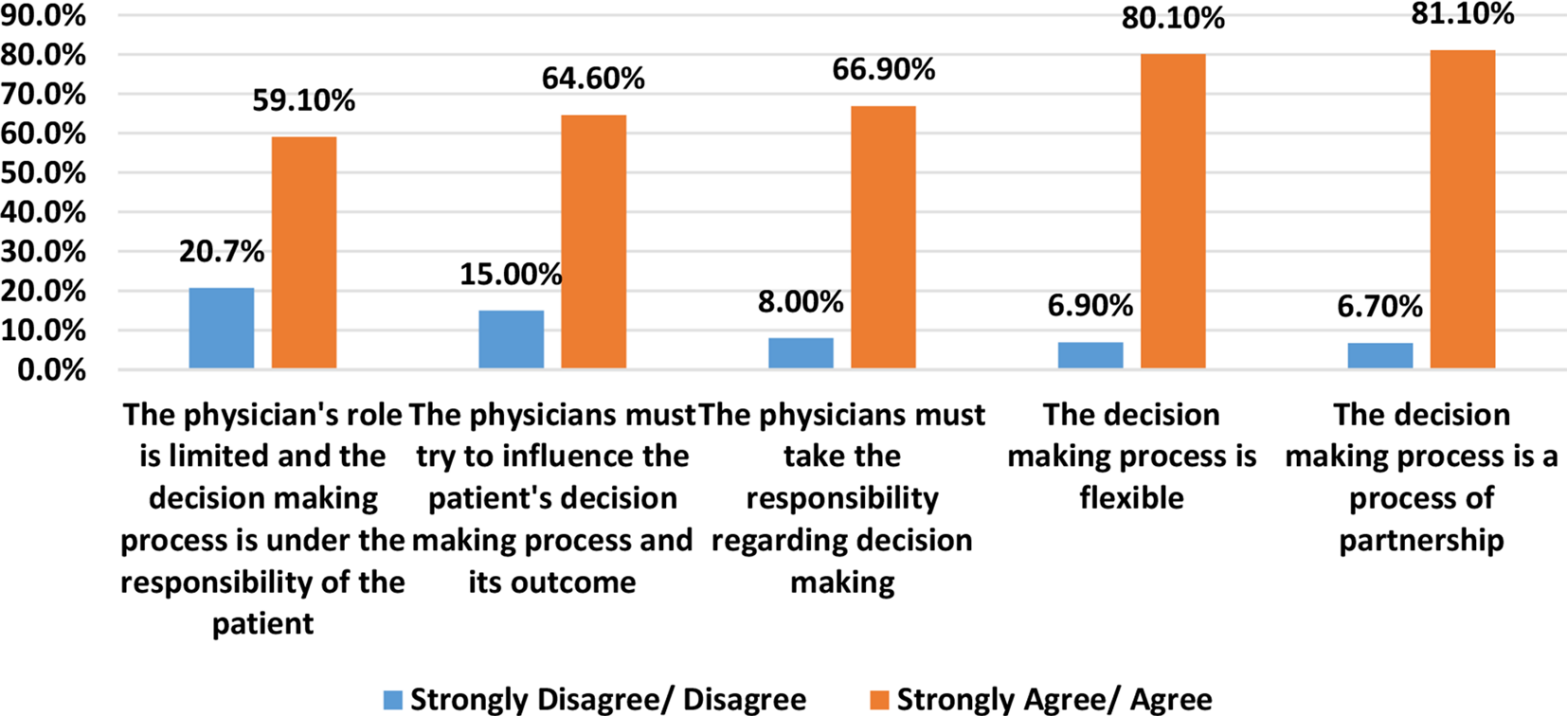
Decision-Making Processes

Most respondents agreed or strongly agreed that decision-making is flexible (80.1%) and is a process of partnership (81.1%); the respondents also tended to agree or strongly agree that they must take responsibility for the patients’ medical decisions (66.9%); that they must attempt to influence the patient’s decision-making and outcomes (64.6%); and that the physician’s role is limited, placing the decision-making responsibility on the patient (59.1%) (Fig. 2).

Some physicians (15.7%) reported making their final decision after consulting other professionals; only 5.5% of the respondents reported making the final decision without any consultation. Decision-making regarding care for ESRD patients was found to be most affected by professional considerations (91.1%), followed by emotional outcomes in relation to the specific case (65.7%). Issues such as legal liability, religious beliefs, self-esteem, workload, and the number of patients were only addressed by half the respondents.

Most respondents reported that they tend to make decisions together with other professionals, including other physicians, nurses, and social workers on the ward (77%), PC staff (64%), physicians outside the ward (56.8%), the hospital Ethics Committee (50.3%), and religious people from the patient’s surrounding (45.6%). Almost half the physicians strongly agreed that collaboration between teams improves patient care efficiency (48.8%), increases job satisfaction (42.1%), and decreases workload (37.8%).

**Fig. 3:**
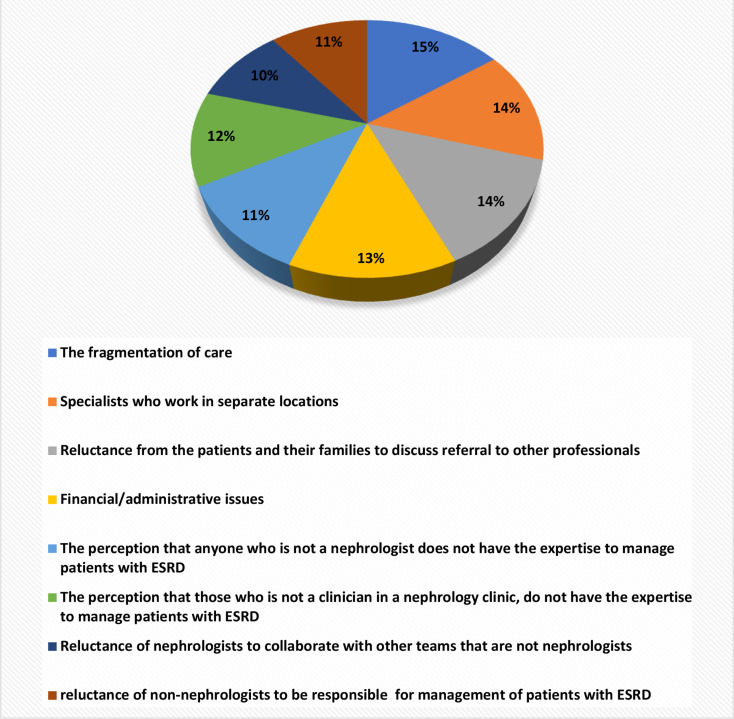
Main Barriers to Collaborations between Teams

Collaborations between healthcare providers were found to be most facilitated by providing consultations within the nephrology clinic (97.2%), yet least facilitated by promoting interpersonal relationships between healthcare providers (81.3%). Obstacles to conducting team collaborations included fragmented care (15%); specialists from different locations (14%); the patients’ and families’ reluctance to discuss referrals to other professionals (14%); and financial/administrative issues (13%) (Fig. 3).

Regarding physician-patient communications, around 80% of the respondents believed that it is important to discuss all available treatment options with ESRD patients. About two-thirds believed that ESRD patients usually refrain from discussing their prognosis (67.1%), do not fully understand the consequences of withdrawing from dialysis (60.8%), and are prone to depression/anxiety (62.2%) – thereby hindering their decision-making abilities regarding EOL care.

About two-thirds of the respondents also believed that physicians regularly discuss quality-of-life with ESRD patients (66.9%), inquire about their preferred place of dying (61.5%), provide prognostic assessments (63.4%), ask their patients about AD/power-of-attorney, and inquire about their religious and cultural beliefs regarding EOL (57.4%). Finally, 20.1% reported never asking their ESRD patients about AD/power-of-attorney, while 58.6% claimed that they always do so.

### Attitudes towards EOL training and care of ESRD patients

Most respondents reported having undergone training on EOL management and symptom management (68%), EOL-related legal issues (66%), and ADs and PC (62%). About 11% reported having received no special EOL training.

Most respondents stated that they feel comfortable conducting conversations with ESRD patients and their families regarding prognosis, quality-of-life, and treatment options (76.12%), managing PC for patients who have stopped receiving dialysis (73.69%), and assisting them in completing their AD or appointing power-of-attorney (72.94%). They also reported that they had received adequate training in managing and evaluating EOL among ESRD patients (71.86%).

**Fig. 4:**
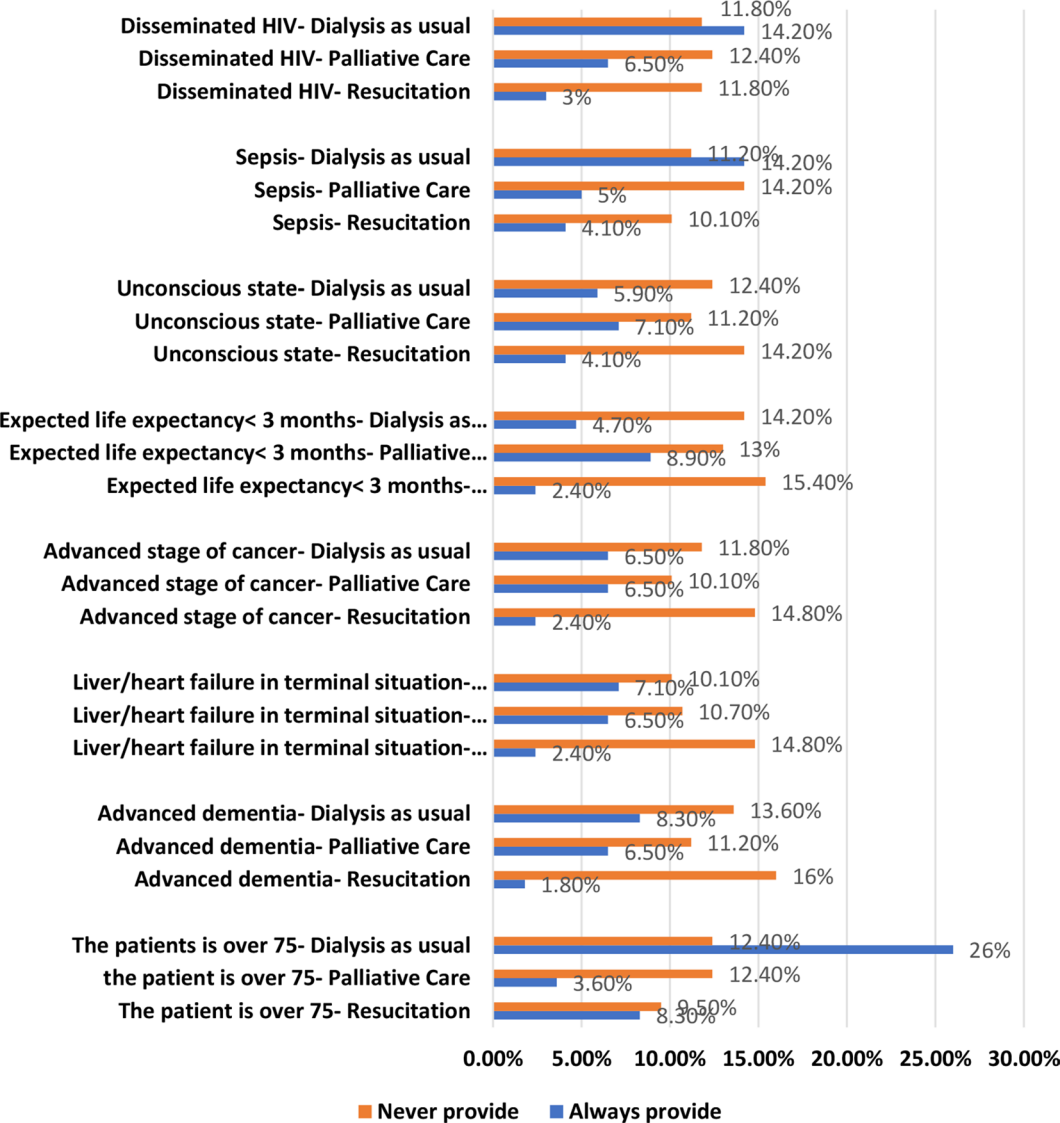
Dialysis as Usual, Resuscitation and Palliative Care in Hypothetical Conditions in ESRD Patients

Few nephrologists reported that they would always provide regular dialysis for patients over 75 (26%), with sepsis (14.2%), or with HIV (14.2%), yet would never do so for patients with advanced dementia (13.6%). PC was found to usually be offered to patients who are unconscious (7.1%) or have a life expectancy of < 3 months (8.9%); yet not for patients with sepsis (14.2%). Finally, few nephrologists reported that they would resuscitate ESRD patients over the age of 75 (8.3%), and would never suggest resuscitation for patients with a life expectancy of < 3 months (15.4%) or with advanced dementia (16%) (Fig. 4).

**Fig. 5:**
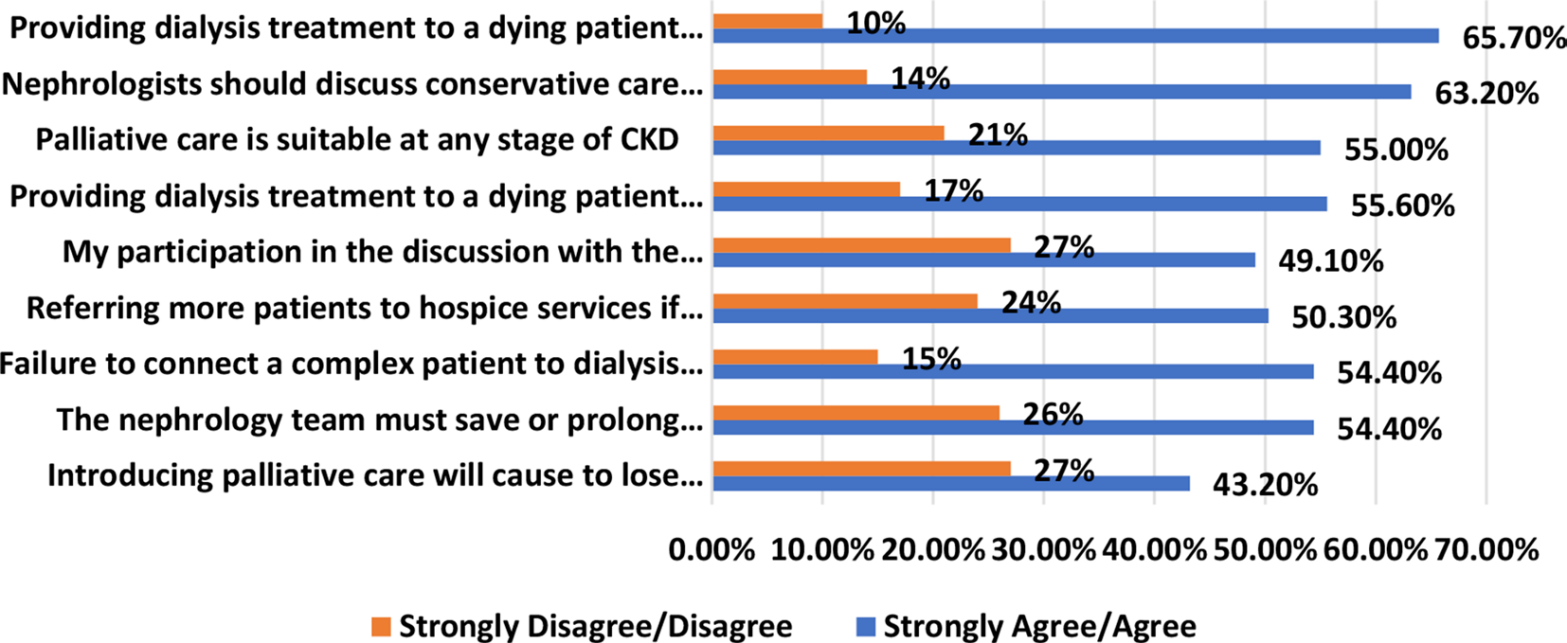
Respondents’ Beliefs regarding EOL Situations

Many respondents (65.7%) agreed or strongly agreed that providing dialysis to dying patients is equivalent to other life-saving treatments, which in Israel (according to the Dying Patient Act 2005, cannot be terminated, but may not be renewed. Overall, between two-thirds of respondents agreed that nephrologists should discuss CC or AD with ESRD patients, and refer them to PC (Fig. 5).

### Relationships between SDM styles and treatment decisions

According to our first hypothesis, interpretive and informative nephrologists, and those who work in public/government dialysis clinics, will tend to support CC, while focusing on the patients’ engagement, values, autonomy, and quality-of-life. This hypothesis was partially confirmed by the findings in this study. Informative nephrologists were found to prefer the value of autonomy over health (*P* < 0.00), were more in favor of conservative treatment – thereby referring to the concept of quality-of-life, and used less invasive treatments compared to other nephrologists (*P* = 0.02). Moreover, informative nephrologists focused more on values of autonomy and quality-of-life than on the patients’ health when making clinical decisions (*p* < 0.0001).

According to the second hypothesis, paternalist and older nephrologists will tend to prioritize their patients’ health over their autonomy, and will be in favor of initiating dialysis as a measure of success. This hypothesis, however, was not confirmed, as the research sample did not include nephrologists who typically act in accordance with the paternalistic decision-making style. Instead, 47% of respondents reported applying an interpretive decision-making style, and 53% reported applying an informative one. Additionally, no significant correlations were seen between age and the tendency to promote the patient’s health and initiate dialysis rather than promoting their autonomy. Finally, nephrologists from the public/government organizations were not found to prefer CC to more aggressive care compared to those who work in private institutions.

## Discussion

### Providing EOL care for ESRD patients

This study investigated the extent to which nephrologists in Israel pursue an SDM decision-making model when providing EOL care for ESRD patients. Specifically, the study explored different patterns of decision-making processes that can be applied in EOL situations, and that stem from a variety of reasons. First, the categories describing EOL situations are not clear-cut (as seen in Sect. 4.3 above). Second, a gap may exist between the respondents’ declared and actual actions, allowing for a wide range of options and fluctuations. Third, the research sample was diverse in terms of age, experience, workplace, and other variables. Such a dynamic approach to EOL care within nephrology has been previously observed in studies conducted outside Israel [39, 50, 52]. Moreover, nephrologists may transition between models and approaches in terms of the degree of SDM and communications between the various parties [49].

Davison and colleagues (2006) [53] found that young nephrologists from Canada reported stopping dialysis more frequently than nephrologists from the USA, especially in cases of severe dementia (as seen in this study), although they practiced medicine in units that were less likely to have policies on dialysis withdrawal. Additionally, unlike a previous study [54], no relationships were seen between the physicians’ age and their referring ESRD patients to PC. This may be due to the relative high importance that is awarded to sanctity of life in Israel, compared to other Western countries [33, 52].

### Cultural explanations

In this study, some (albeit few) respondents were willing to initiate dialysis or resuscitation for patients over the age of 75 without additional co-morbidities. The desire to offer optimal care for the elderly exemplifies the Israeli ethos to care for elderly patients at almost any cost [55], unlike other societies where nephrologists are less inclined to begin renal-replacement therapy in such patients [56]. More generally, it represents a tendency among physicians in Israel to preserve life, even with poor quality-of-life [34, 57], which reflects a shift in the attitudes of healthcare professionals in Israel towards PC [58]. Yet unlike our expectations, and as demonstrated in a study conducted in Germany on the opinions and practices of head physicians in renal centers [59], no tendency to offer CC was seen in public institutions compared to private ones.

### Nephrologists’ decision-making styles

One of the most dominant findings of this study is that no respondents from the research sample conveyed that they employ a paternalistic decision-making style. Moreover, about 80% of the respondents believed that it is important to discuss all available treatment options with ESRD patients. These findings are in line with Yagil & Medler-Liraz, (2015) [60], who found that family physicians in Israel use various tactics for providing guidelines, maintaining their professional identity, and involving patients in the decision-making process. Yet these findings are in contrast to a study by Einav and colleagues, where intensive-care physicians in Israel were found to be more paternalistic than their counterparts in the USA [61]. It may be that nephrologists give more weight to their patients’ preferences than other physicians.

In this study, almost 60% of the participating nephrologists stated that it is the physician’s responsibility to influence the patient’s decision-making, with most applying interpretive and informative decision-making styles. Moreover, the study shows that one main factor that impacts the decision-making process is the possible emotional outcome of the nephrologist-patient discussions and communications on all parties involved, especially the patients and their families. To reduce this burden, nephrologists may implement different decision-making styles – such as paternalistic, informed, or SDM. They may also avoid conducting such direct discussions, by simply convincing their patients to undergo dialysis. Alternatively, they might transfer the decision-making responsibility to the patients, or conduct time-limited trials of dialysis [18].

The study also reveals that informative nephrologists prefer the value of autonomy over health, are more in favor of CC, and use less invasive treatments –compared to interpretive nephrologists. In a qualitative study by Ladin et al. (2018) [40] on decision-making in nephrology, five themes were found to differentiate between the four decision-making styles: [1] patient autonomy; [2] engagement and deliberation; [3] the influence of institutional norms; [4] the importance of clinical outcomes; [5] and the physician’s role. Paternalist nephrologists were found to view dialysis initiation as a measure of success, while advocating for dedication to patients and commitment to treatment. On the other hand, interpretive and informative nephrologists were found to focus on patient engagement and quality-of-life, while aligning the treatment with the patients’ values. Interpretive nephrologists were also found to place an emphasis on trust and on the understanding of their patients’ preferences. Similar to the current study, informativists perceived patient autonomy as the most important component in the decision-making process, enabling patients to enjoy the life they have left and live it with dignity. Yet only one-third of the informative and interpretive physicians in their study offered CC to their patients – unlike the findings of the current study.

### Content of nephrologists’ communications with ESRD patients

In the current study, about two-thirds of the respondents reported that they always provide their patients with prognostic assessments, discuss quality-of-life with them, inquire about their preferred place of death, and ask about their religious and cultural beliefs regarding death and dying. These findings are in line with the guidelines issued by the American Renal Physicians Association, whereby implementing SDM is recommended, as a means for reaching shared understandings and agreements based on common ground [21] as well as with studies conducted outside of Israel, where physicians reported a more direct involvement of patients in the decision-making process [62, 63]. These findings, however, are contrary to a recent study where Israeli healthcare professionals conveyed their hesitance about engaging patients in SDM [64]. Future research should gather more empirical and objective data on the topic, as a means for further understanding the practice of SDM, for example, through surveying patients or analyzing videotape consultation during such processes [65, 66].

### Patients’ perspectives on SDM

Indeed, examining the patients’ perspectives on SDM is of great importance [67]. Increased satisfaction with their received healthcare services and decreased anxiety were seen among patients who were involved in their treatment-related decisions through participation in educational programs related to their specific illness (where a range of aspects were addressed, such as symptoms, clinical outcomes, and the impact of the disease on their lives). They also reported increased knowledge, adherence to treatment, physical outcomes, efficient utilization of health services, and decreased rates of hospitalization [68].

In a study that examined decision-making in ESRD patients, 27% of the participants reported that they made their own medical decisions alone, 24.5% shared the responsibility, and 48.4% relied on their healthcare providers. Older patients reported a more passive role and greater reliance on their healthcare teams, compared to younger patients [43, 62]. Another study found two competing views on decision-making among patients. According to the first view, patients reported a lack of understanding regarding SDM, including limited familiarity with the concept, low acceptance of this process, insufficient information, and lack of autonomy within familial relationships. Yet according to the second view, patients reported that healthcare professionals advocate for SDM, conservative treatment, and EOL care, thereby facilitating advanced decision-making among patients [65]. A study from the USA further revealed that nephrologists are trained to enhance SDM in dialysis-related decisions for older patients with life-limiting ESRD, using a best-case/worst-case decision-making tool (i.e., life with dialysis and life without dialysis). Patients and family members who were exposed to this tool reported that it allowed them to deliberate about the treatment options, anticipate what life with dialysis might be like, and prepare them for the future. Moreover, patients who were exposed to this tool supported SDM because they had been given options [69].

In a recent study, younger nephrology patients were found to value autonomy and their current lives, while older patients tended to focus on “the rest of their lives,” spending time with their families, and preparing for death. The study presents a conceptual model for how older patients decide whether to choose dialysis or CC, based on the following three concepts: [1] reflecting on treatment options in relation to physical frailty and mental health; [2] confronting difficult decisions by considering the need to receive and manage clear information, rather than burdening the family caregivers; and [3] maintaining hope, by choosing to live the rest of their lives with peace and dignity, knowing that they may not have long to live [70].

In an Israeli study on the attitudes of ESRD patients towards dialysis and transplantation, priority was given to younger patients, who were perceived as having better life expectancy than older patients, as well as other improved physical prospects [71]. However, data is lacking on patients’ perspectives and experiences regarding SDM.

Studies show that patients in Israel wish to be involved in treatment decisions [42, 43] and in SDM processes [72], and that in general, the Israeli public is ready for increased engagement and information regarding their healthcare [73]. Indeed, progress can be seen, for example, through the increasing engagement of patients with chronic diseases and their families, following patient centered care (PCC) and SDM approaches, as well as public policies [64]. However, most patients do not feel ready to be involved in their consultations [43], and prefer decisions to be made by their physicians [74]. Some patients are reluctant to engage in active decision-making, since they believe that they lack adequate medical understanding and knowledge of up-to-date studies. As such, patients vary in the degree to which they wish to take part in the decision-making process regarding their own health [74]. To achieve further progress in PCC and SDM in Israel, one healthcare organization encourages patients to use the “Ask Me Three” tool, that helps them ask their physician specific questions about their condition, what should be done to treat it, and why that course of treatment is important or correct [64]. Yet, there is still room for improvement in implementing and maintaining PCC and SDM [44]. To enhance SDM in Israel, data must be collected, analyzed, and stored in an organized and accessible manner; adequate healthcare infrastructure should be provided; awareness of this process must be increased; and decision-making tools should be developed and then implemented by both medical teams and patients [64, 75].

### Making treatment choices at EOL and advance directives

In the current study, a clear divide was found between nephrologists who never ask patients with ESRD about their AD/power-of-attorney compared to those who always do so. This finding contradicts our expectations, whereby nephrologists bear the responsibility for guiding patients on such matters [53]. Treatment choices near EOL are not simple or predictable; rather, they are uncertain and complex. The aim of advance care-planning is to support patients when they lack decisional capacity and are unable to understand or share their medical preferences in the future. Yet, as studies and practices show, AD do not suffice. In some cases, their existence may even inhibit discussions, leading to *present* decisions being made based on documents that are supposed to be used in the future [76, 77]. Empathic listening may be the key to alleviating the patient’s uncertainty in making decisions regarding EOL care. It can help patients feel less overwhelmed, tolerate their prognosis with greater ease, and increase their adherence to treatment [78].

### Professional, emotional, cultural, and organizational aspects of EOL decision-making

Finally, when reviewing factors that impact decision-making for ESRD patients, this research found both professional considerations and emotional consequences to be most influential – similar to other studies [79, 80]. Moreover, the current study found that the patients’ religious beliefs play a role in the decision-making of about half the nephrologist who completed the questionnaire. According to previous studies, maintaining life-support is a cultural value that is embedded in religion [81, 82]. Our study also found that the physicians’ decision-making practices were impacted by their legal responsibility, workload, and the number of patients that they tend to treat within a given period. Our findings are also in line with another study, where factors that were central to EOL decision-making included the physicians’ professional experience, legal issues, and patient-related factors, such as their wishes and prognosis, and the requests of the patients and their families. The least influencing factors in decision making were hospital-related variables, such as specialization, medical hierarchy, and time pressure [83].

### Policy implications

The varied findings presented in this study offer a number of important policy implications regarding care-provision and decision-making for ESRD patients. These include the following:

- The findings of this study indicate that nephrologists in Israel are not paternalistic. Theoretically, they acknowledge the important role of the patients within the SDM process. Yet, in practice, they do not always involve the patients, with whom their communications are rather limited. To minimize this gap, healthcare professionals should be encouraged to increase their patients’ awareness of SDM. Moreover, healthcare organizations should also develop and offer educational programs, tools, and modalities – especially suited to recurring nephrology patients, who could learn more about their illness and its impact on their lives and on their clinical condition. Doing so will enhance the patients’ knowledge and willingness to engage in SDM [74]; improve their ability to ask questions [44]; increase their desire to deliberate about treatment choices and dialysis [69]; support decisions related to kidney-failure treatment modalities [84]; improve adherence to treatment; and advance their substantial participation in SDM [68].
- The study found that the majority of nephrologists perceived SDM as a process of partnership between multi-disciplinary teams. Most respondents claimed to consult other professionals, especially nephrologists and nurses, yet not physicians of other specialties, nor social workers or PC staff. Establishing and maintaining co-working and consultation conditions between nephrologists and other professionals could contribute to SDM processes and outcomes.
- As many of the nephrologists do not ask their patients about AD or power-of-attorney, physicians and nurses who provide care to ESRD patients should be encouraged to hold such conversations with their patients, while providing them with the necessary knowledge and assistance for completing the required forms. Doing so will greatly facilitate SDM in future encounters with these patients. Moreover, increasing the percentage of ESDR patients who tend to such issues in advance should be a key target in medical institutions; this could also serve as an additional factor in the accreditation process of these institutions.
- More generally, in order to improve SDM processes regarding ESRD patients, trainings on EOL care and ethics should be conducted regularly, for both nephrologists and nurses. Such programs could better equip healthcare providers for conducting SDM processes, while improving their communication skills required for SDM, emphasize the importance of listening, and showing empathy to the patient. Such trainings could also include SDM simulations, and offer an SDM checklist (which could be created using artificial intelligence). This would help healthcare providers offer greater support for their patients when making decisions about their treatment, allowing them to weigh their options in a more informed manner [85].
- Finally, the ISNH should consider issuing guidelines – similar to those published by the American Renal Physicians Association. Doing so will convey a clear statement as to the importance of implementing SDM when treating ESRD patients. Combined with the recommendations presented above, this top-down approach will help internalize the effective practice of SDM in nephrology units across Israel, and in turn, may inspire additional medical fields and practices to also do so.

### Strengths and limitations of the study

- This study focused on nephrologist-ESRD-patient SDM. As such, generalization of these findings to other physicians and fields should be made with caution. However, as a very high percentage of nephrologists responded to the survey, the findings are highly indicative of the views and attitudes of the researched population.
- The topic of SDM was only examined from the physicians’ perspective, not the patients’ perspective. It may be argued that the design and findings of this study reinforce a paternalistic view of the clinician-patient relationship, which assumes that decisions should be made solely or chiefly by physicians [86]. Yet no paternalistic pattern of SDM was seen among the surveyed nephrologists in the current study. Future research could benefit from examining the perceptions of nephrology patients, in addition to physicians in this field, for means of comparison and for identifying discrepancies between the attitudes of these two populations. Regardless, our report of this finding in itself is strong enough to refute this concern. Additionally, the study did also examine and report other findings regarding the patients’ attitudes and capabilities, albeit indirectly, through the nephrologists’ input.
- Certain bias may have occurred in the data collection, as the questionnaire employed in this study was relatively long; as such, certain groups of respondents – such as those who are greatly in favor of or against SDM – may have invested in completing this survey. Yet the 169 questionnaires that were completed in full provide a large sample size in itself, and also represent about 60% of the entire nephrologist population in Israel, which is considered a desirable rate [87]. Additionally, as a cross-sectional study, this research does not claim to represent the entire population.
- Not only does this study describe the actual practices of SDM in EOL nephrology care; it also examines important associations between SDM and the nephrologists’ attitudes towards ethics during EOL care, thereby providing important evidence for further examining the practice of SDM in Israel.

## Conclusions

Nephrologists in Israel only partially pursue the SDM model. They tend to ask patients about their AD or power-of-attorney, and inquire about their religious and cultural beliefs regarding EOL. Yet when providing care to ESRD patients with complex cases, these physicians may decide to take sole responsibility for the decision-making, regarding withholding or withdrawing from dialysis, for example, or when referring patients to PC. Additionally, nephrologists in Israel are not paternalistic, and are aware of the patients’ important role in the SDM process. However, enhancing SDM in nephrology programs in Israel is highly recommended – with an emphasis on the importance of listening to patients, conveying empathy, enhancing patient knowledge, and improving communications between patients and healthcare providers. Doing so will be beneficial for all parties involved.

## Annex 1. The Questionnaire

The questionnaire that was comprised for this study included 92 items, most of which were close-ended questions, where the respondents were asked to rate each item on a Likert-like scale; 12 items were open-ended questions. The questionnaire mainly focused on existing items from validated questionnaires that have been published in the literature, which were then translated into Hebrew. The questionnaire was comprised of four sections. *The first section* included 12 biographical questions, such as age, marital status, work experience, and type and location of workplace. *The second section* of the questionnaire was adapted from the Clinician Perspectives on Palliative Care in Kidney Disease Questionnaire [28], and included seven items about the respondents’ education and training in EOL care and decision-making. Two items were close-ended questions that aimed at assessing the participants’ medical training (items 13, 14). Five items were related to the respondents’ training in EOL care management and providing patients with assistance in preparing their AD or appointing power-of-attorney (items 15–19), which the respondents were asked to rate on a Likert-like scale, from 1 (strongly disagree) to 5 (strongly agree). For example, “I feel confident in managing PC for patients who have stopped receiving dialysis and their families,” or “I feel comfortable holding conversations with ESRD patients and their families regarding the prognosis.” The first author of this paper received the questionnaire and related input from the first author of the original questionnaire via email [28]. The questionnaire had undergone validity tests, including extensive pilot testing that entailed the completing of the questionnaire by experts in the field, following by their submitting a survey about the questionnaire comprised of both open-ended and multiple-choice questions.

*The third section* of the questionnaire was in line with the conceptual framework of this research [48–50, 88]. These 40 items, which were organized into three groups of questions, examined the respondents attitudes and practices regarding SDM, based on the key elements suggested by these theoretical models: [1] The first group of the questions (items 20–31), which were adapted from Makoul and Clayman (2006) [49], assessed whether the respondents define the problem for the patient during the decision-making process, for example, or whether they offer the patient free choice regarding the proposed treatment; [2] The second group of questions (items 32–36) were adapted from Charles and colleagues (1999) [50] and Makoul & Clayman (2006) [49]. These items asked respondents to rate their level of agreement with various statements, on a Likert-like scale from 1 (strongly disagree) to 5 (strongly agree), such as the physician’s ability to influence the patient’s decision-making process and its outcome, and whether they regard the decision-making process as a process of partnership; [3] The third group of questions (items 37–38), which were adapted from Charles and colleagues (1999) [50], aimed at assessing who the physician tends to involve when making decisions regarding ESRD patients using close-ended multiple choices questions. The following questions (items 39–41, 44–45) were adapted from Ceckowski and colleagues (2017) [29]. These items referred to decision-making processes with regards to several situations, such as an ESRD patient who suffers from depression, or whose family has limited understanding of PC and hospice care. Items 42–43, 46–51, 52–59 of the questionnaire were adapted from Metzger and colleagues (2021) [28] and were aimed at assessing the extent to which respondents provide prognostic assessments, for example, and whether they discuss all treatment modalities with their patients. For these items, the respondents were asked to choose their rate of agreement (on a scale of: *never*, *rarely*, *sometimes*, *often*, and *almost always*.) For other items, the respondents were asked to provide specific input, such as “What is the most prominent obstacle” (Item 53) or “What percentage of your patients have prepared advance medical directives?” (Item 54).

Finally, *the fourth section* of the questionnaire included 33 items that referred to EOL care for patients with ESRD, and to the nephrologists’ attitudes towards such care, based on Lunney et al.‘s (2002) categorization of EOL care [89]. Items 60–62 included open-ended questions, which asked respondents to address issues such as a possible age limit for withdrawing patients from dialysis, their perceived role of PC, and the complexity of providing care for ESRD patients. Items 63–70, which were adapted from Hong and Colleagues (2021) [25], aimed at assessing how nephrologists would act based on a list of care alternatives in various hypothetical scenarios, for example, dialysis as usual, referral to PC, and resuscitation when needed. For example, when the patient is over 75 years old; has a life expectancy of up to three months; or has cancer. The respondents were asked to rate each item on a Likert-like scale, from 1 (almost never true) to 5 (almost always true). Item 71 was adapted from Fung and Colleagues (2016) [19] and explores what best describes the physician’s decision-making process based on a close-ended multiple-choice question. Items 72–73 aimed at assessing workplace practices with regards to SDM [90]; items 74–75 were adapted from Metzger and colleagues (2021) [28] and aimed at exploring the reasons for referring ESRD patients to PC.

The next items [76–83], which were adapted from Hong and colleagues (2021) [25], aimed at assessing how often clinicians meet with ESRD patients in the various scenarios suggested above. The respondents were asked to rate each item on a Likert-like scale, ranging from 1 (less than five patients per month) to 5 (more than 20 patients per month). Finally, items 84–85, which were also adapted from Metzger and colleagues (2021) [28], explored the respondents’ attitudes towards the consequences of referring patients to PC, based on a Likert-like scale from 1 (strongly disagree) to 5 (strongly agree). Regarding Items 86–92, Item 86 was adapted from Ceckowski and colleagues (2017) [29] and aimed at assessing the respondents’ willingness to refer patients to a hospice if they can undergo hemodialysis. Items 87 and 92 were adapted from Perry et al., 1996 [91], in an aim to assess the respondents’ attitudes towards providing care for EOL patients. Item 88 was aimed at assessing whether the nephrology staff discusses CC with patients in advanced chronic kidney disease. Items 89–91 were adapted from Hong and Colleagues (2021) [25], aimed at assessing respondents’ beliefs, for example, regarding the nephrology staff’s role in saving or prolonging the lives of ESRD patients, on a Likert-like scale from 1 (strongly disagree) to 5 (strongly agree). The research questionnaire is in Hebrew and can be re-used by the authors’ permission upon request.

## Annex 2: The Conceptual Framework

**The conceptual research framework** combines the following theoretical SDM models, each comprised of three sections: [1] practical SDM steps; [2] SDM categories; and [3] types of decision-making (Fig. 1).

**The three practical SDM steps** for clinical practice include *a choice talk*, where physicians ensure that the patient is presented with reasonable care/treatment options; *an options talk*, where physicians must provide patients with additional information about the various options; and *the decision talk*, where physicians must consider the options and preferences and then choose the optimal option for the patient [48].

**The three SDM categories** include *essential SDM elements*, comprised of defining the medical issue, presenting an option, discussing benefits, risks and costs, exploring patient values and preferences, discussing patient ability and self-efficacy, exploring the doctor’s knowledge and recommendations, checking the patient’s understanding, making or deferring decisions, and arranging follow up; *ideal SDM elements*, comprised of introducing unbiased information, defining roles, presenting evidence, and reaching a mutual agreement; and *general SDM qualities*, comprised of deliberating and negotiating, applying a flexible and individualized approach, exchanging information, involving at least two people, finding middle ground, acting with mutual respect, seeking a partnership, enhancing patient education and participation, and deciding on the process/stages [49].

The third and final model of the conceptual framework for this research is offered by Charles and colleagues [50], who in revising an earlier framework (1997) propose **three types of decision-making** in relation to choosing the patient’s recommended course of treatment. The first type is *paternalistic*, where physicians have full authority to determine the treatment that is to be implemented. The second type is *SDM*, where the physician and patient jointly discuss and choose the treatment that is to be implemented. Finally, the third type relates to informed decision-making, where the patient has the exclusive authority to make decisions regarding their own treatment.

## Data Availability

The datasets used and/or analyzed during the current study are available from the corresponding author on reasonable request.

## Abbreviations

AD: Advance directives
CC: Conservative care
EOL: End-of-life
ESRD: End-stage renal disease
PC: Palliative care
SDM: Shared decision-making
